# Brain Structural Effects of Antipsychotic Treatment in Schizophrenia: A Systematic Review

**DOI:** 10.2174/1570159X13666150429002536

**Published:** 2015-07

**Authors:** Roberto Roiz-Santiañez, Paula Suarez-Pinilla, Benedicto Crespo-Facorro

**Affiliations:** Department of Psychiatry, University Hospital Marqués de Valdecilla, School of Medicine, University of Cantabria-IDIVAL, Santander, Spain, CIBERSAM (Centro Investigación Biomédica en Red Salud Mental), Spain

**Keywords:** Antipsychotic, longitudinal studies, schizophrenia, structural magnetic resonance imaging.

## Abstract

The findings about the progressive brain changes in schizophrenia are controversial, and the potential confounding effect of antipsychotics on brain structure is still under debate. The goal of the current article was to review the existing longitudinal neuroimaging studies addressing the impact of antipsychotic drug treatment on brain changes in schizophrenia. A comprehensive search of PubMed was performed using combinations of key terms distributed into four blocks: “MRI”, “longitudinal”, “schizophrenia” and “antipsychotic”. Studies were considered to be eligible for the review if they were original articles. Studies that examined only changes in brain density were excluded. A total of 41 MRI studies were identified and reviewed. Longitudinal MRI studies did not provide a consistent notion of the effects of antipsychotic treatment on the pattern of brain changes over time in schizophrenia. Overall, most of the included articles did not find a linear relationship between the degree of exposure and progressive brain changes. Further short- and longterm studies are warranted to a better understanding of the influence of antipsychotics in brain structural changes in schizophrenia and also to verify whether first and second generation antipsychotics may differentially affect brain morphometry.

## INTRODUCTION 

Schizophrenia is a common chronic and disabling brain disorder. The nature of the disease process remains obscure. “Imaging evidence indicates the consistent association between brain structural abnormalities and schizophrenia” [1]. These brain alterations are already present prior to the development of the disease [2, 3]. Although the pattern of brain changes over time is still under debate, a recent meta-analytic study suggests that these brain alterations may progress over time [4]. “Progressive brain changes could be associated with neurodegenerative or neurotoxic processes” [5], “attributed to a plastic adaptation of the brain to the environment” [6], possible neurotoxic effects of hyper-dopaminergia as well as interactions with the glutamatergic system [7], oxidative stress [8], and related to exposure to pharmacological treatment, as antipsychotic drugs [9].

“The potential effects of antipsychotic medication on brain structure might represent a key factor in understanding brain changes in psychosis” [10]. Therefore, the investigation of the “effect of antipsychotic drugs on progressive brain tissue loss has attracted much interest in the field. The therapeutics and deleterious actions of antipsychotic drugs could be mediated, in part, by their cellular effects and consequently also linked to morphometric changes” [11]. Moreover, “brain volume abnormalities appear associated to the outcome of the illness” [12]. First and second generation antipsychotics (FGAs and SGAs) “may exert neurotrophic, neurogenetic and neuroprotective effects differentially” [13]. Animal and cell biology studies have demonstrated the differential effects of FGAs and SGAs on cellular morphology and brain growth factors. And SGAs seem to have differential long-term neuroprotective actions compared with haloperidol [14, 15]. The goal of the current article was to review longitudinal neuroimaging studies exploring the impact of antipsychotic drug treatment on progressive brain changes in schizophrenia. 

## METHODS 

We systematically reviewed the literature to identify journal articles reporting antipsychotic effects on brain morphometry detected with neuroimaging techniques. Studies were searched in PubMed from January 1994 to July 2014, using combination of key terms distributed in four blocks: “Magnetic Resonance Imaging (MRI)”, “schizophrenia”, “longitudinal” and “antipsychotic”. The identified publications were hand-searched in order to select longitudinal neuroimaging studies considering the effects of antipsychotic medication. Studies were considered to be legible for the current review if they met the following inclusion criteria: 1) they were original publications written in English in a peer-reviewed journal; 2) they were longitudinal structural MRI studies; 3) they included brain volumes or cortical thickness or surface area variables; 4) they examined the effects of antipsychotic medication on brain structure; 5) patients were diagnosed with schizophrenia spectrum disorders. Studies were excluded for any of the following reasons: 1) other designs, such as case-reports, series of cases, etc; 2) non-original studies, including editorials, prefaces, brief communications and letters to the editor, literature reviews or meta-analyses; 3) Results no described the effect of antipsychotics in brain changes; and 4) studies examined only changes in brain density. “Interpreting data across Voxel-Based Morphometry (VBM) studies is a problem because there are a large number of factors with studies using different degrees of smoothing and different registration and segmentation algorithms that can vary and influence the results” [16].

Each article was read in its entirely, and data elements were then extracted and entered in a customized database. Data were extracted from the source documents independently by two investigators (RRS, PSP). The information collected from each article included the following: 1) authors; 2) publication year; 3) ethical approval; 4) sample size; 5) type of patients (chronic or first episode of psychosis (FEP)); 6) form of analysis completion/intention to treat; 7) period of follow-up; 8) antipsychotic drugs and doses; 9) MRI analyses type and software; 10) brain structures involved; and 11) main findings.

The literature investigating the effects of antipsychotic treatment on progressive brain changes includes different study designs: 1) longitudinal studies evaluating the effect of different antipsychotic treatments; 2) longitudinal studies evaluating the impact on brain morphology of a switch from FGAs to SGAs; 3) longitudinal studies conducted in healthy controls and medicated patients evaluating the association between progressive brain changes and antipsychotic exposure in patients. Therefore, to provide a better analysis, the studies were grouped into three categories: i) effect of different antipsychotic treatments; ii) effect of medication switch; and iii) association with the amount of antipsychotic exposure. 

## RESULTS

**Fig. (1):**
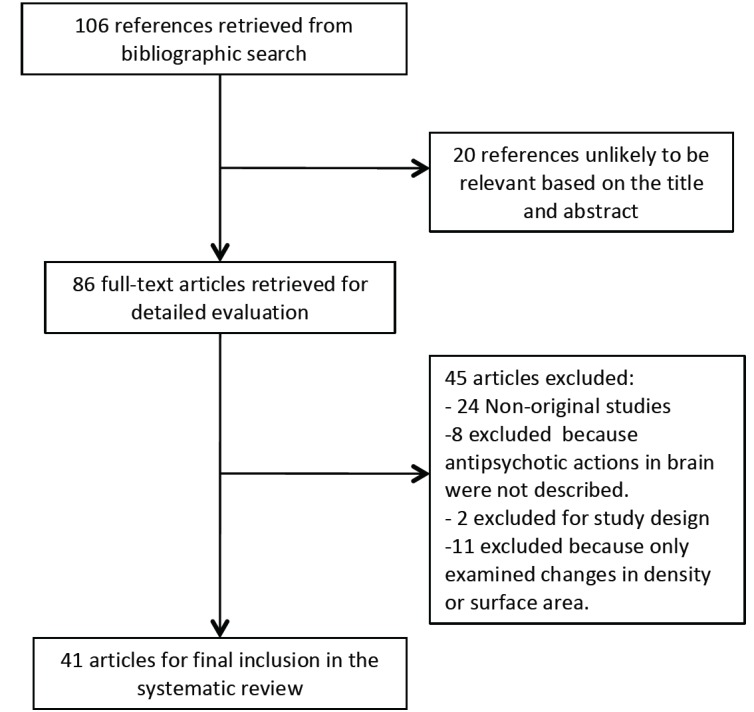
Flow diagram process of study selection.

Electronic database search yielded 106 references. Fig. **1** illustrates the flow diagram of the study selection process. After the selection process, we identified 41 longitudinal studies that satisfied the inclusion criteria (Table **1**). Twelve studies compared the effect of different treatments on brain morphometry, 31 were focused on the association between progressive brain change and antipsychotic medication exposure, and two analysed the effect on brain morphometry of an antipsychotic medication switch. Four studies were included within more than one group.

### Association with Antipsychotic Exposure

There were thirty MRI longitudinal studies that performed correlations or regression analyses to investigate if there is any association between the degree of exposure to antipsychotic drugs and progressive brain changes. Most of the studies (N=20) enrolled patients with an FEP, seven included patients with chronic schizophrenia, and four articles covered a mixed sample of drug naïve FEP and chronic patients. Fifteen articles enrolled patients taking FGAs and SGAs, eleven studies included patients with SGAs, one study investigated patients treated only with clozapine, in two articles patients were taking only FGAs, and in one study the type of medication was not specified.

With regard to gray matter (GM) and white matter (WM) volume, results are disparate. Certain studies failed to demonstrate any significant correlation between progressive GM changes and antipsychotic exposure. No significant correlation between SGA treatment in children and adolescent with early onset of schizophrenia and brain changes over two years was found [17, 18]. In line with these results, after one year and a half, 17 patients with FEP did not show differences in neocortical GM changes when they were divided in two groups: compliance antipsychotic medication and non-compliance treatment groups [19]. In these three studies, patients were only treated with SGAs (measured in chlorpromazine equivalents). Similarly, McClure *et al*. (2008) described an absence of regional GM volume changes after a short period (four months) of SGA exposure in a sample of chronic schizophrenia patients (N=10; mean age: 36.7 years) [20]. In contrast, some other studies have observed significant associations between progressive GM changes and antipsychotic medication treatment. Thus, a loss of GM was observed in 34 FEP patients during the first year of the illness. GM loss was significantly correlated with higher cumulative dosage of antipsychotic medication used during the follow-up period. No interaction with type of antipsychotic (FGA and SGA) was found, but it is of note that only a reduced number of subjects was taking FGAs (N=5) [21]. Gur and colleagues (1995) exploring a sample of 20 FEP patients showed that higher doses of antipsychotics, both FGAs and SGAs, were associated with greater reduction in frontal (r=-0.75) and temporal (r=-0.66) GM volume across time. However, these results were not repeated when chronic schizophrenia patietns were analyzed [22]. Concerning only SGAs, a lengthy intake of olanzapine during five years was correlated with less reduction in GM volume, and even it was related with a subtle GM increment [23]. On the other hand, risperidone treatment increased GM volume in the right and left caudate nuclei and the left accumbens in 11 FEP patients after three months of treatment. In this latter study, it is worth noting that the dose of risperidone (605 mg/day, in CPZ equivalents) was more than twice higher than the usual dose used in the most of neuroimagen studies [24]. In 2005, Molina *et al*. obtained similar results in a longitudinal study during two years. There was an increase in GM in 17 patients using risperidone. Frontal GM was also increased in 12 chronic patients taking clozapine. In this study, clozapine and risperdone also produced a reduction in WM volumen [25]. In 2001, Ho and colleagues performed the largest sample size study included in the present review (N=211) exploring FEP patients over a period of seven years. Patients were taking FGAs, SGAs or clozapine. WM but not GM volumes showed a significant time by treatment interaction. Therefore, patients of the highest dose group had longitudinal WM volume reductions [26]. 

Among the research conducted on the whole brain volume (WBV), results did not show a direct relationship between changes and drug exposure. Three studies based in children and adolescents with childhood onset of schizophrenia did not find any association between any treatment features with SGA, such as specific drug or dosage, and WBV. Patients were followed during two [17, 18] and three years [27]. In 2003, Ho and colleagues conducted a longitudinal study of 171 weeks in 72 patients with a recent onset of schizophrenia, taking FGAs and SGAs. They reported no significant effects between any of the studied brain regions, including WBV, and cumulative antipsychotic doses, treatment duration, or percentage of time treated with FGAs, SGAs or both types of drugs together [28]. One study based on drug-naïve FEP patients found independence between antipsychotic treatment (FGA, SGA) and changes in straight gyrus [29]. Moreover, there was no association between annual change insular cortex and cumulative days of inpatient treatment per year during two and four years of follow-up [30]. 

The studies investigating the relation between antipsychotics and cortical thickness changes have also shown mixed results. In 20 adult patients (mean age = 31.9 years), frontal and temporal cortical thickness was not associated with interval antipsychotic medication use, both FGAs and SGAs in CPZ equivalents, after two years [31]. However, in 2013, Goghari and colleagues (2013) demonstrated that short-term atypical treatment (eight weeks) with risperidone or quetiapine increased prefrontal cortical thickness in 19 FEP drug-naïve patients (mean age = 18.9 years) [32]. In 2011, Van Haren observed that higher cumulative intake of FGAs “during a five-year scan interval was associated with more pronounced cortical thinning, whereas higher cumulative intake of SGA medication was associated with less pronounced cortical thinning” [33] in long-term schizophrenia patients (mean age = 32.2 years).

Several studies have addressed the issue of antipsychotic medication effects on basal ganglia morphometry. Tauscher-Wisniewski and colleagues found a decline in caudate volume over time in a sample of FEP patients and healthy controls but it was independent of medication [34, 35]. However, other two studies showed a caudate enlargement during the course of treatment with FGAs and SGAs in young drug-naïve schizophrenia patients [36, 37]. This increment was associated with greater amounts of antipsychotic medication received by patients before the first scan [36]. In chronic schizophrenia patients (mean age=31.6 years), treatment with olanzapine seemed to increase the caudate volume after six months [38]. Similarly, Frazier and colleagues (1996) showed a caudate enlargement in patients with childhood onset of schizophrenia who were taking FGAs during two years. In this sample, caudate volume decreased in the subgroup of patients who were changed to clozapine [39]. McClure and colleagues (2008) found no longitudinal changes in caudate volume after brief periods of SGA exposure [20]. On the other hand, in 2005, Taylor and colleagues reported an “increase in left striatum that was not associated with drug treatment, but with a reduction of positive symptoms” [40] over the first month. The sample included patients with an FEP (mean age= 34.7 years) treated with FGAs and SGAs and followed during four weeks [40]. Ebdrup *et al*. (2011) reported that high doses of quetiapine may attenuate the striatal volume loss over time in FEP patients. When patients were compared to healthy control subjects, this volume loss was more prominent in those patients treated with low dose of quetiapine and less apparent in those treated with higher doses of quetiapine. “Post-hoc analyses revealed that the volume loss was pronounced in caudate and putamen nucleus, but not in accumbens nucleus” [41]. They also described that hippocampal volume loss appeared more pronounced in the group of patients treated with higher quetiapine doses [41]. When treatment discontinuation effects were investigated, it seemed to reverse effects of SGAs. A decrease in the volume of accumbens and putamen nucleous occurred in eight FEP patients who discontinued the treatment with SGAs. Conversely, an increment in those volumes was found in patients who did not discontinue the treatment, during one-year follow-up [42]. Gender effect of SGAs on progressive caudate changes has been also considered. SGAs treatment had a negative correlation (r= -0.74) with caudate volume in females, and a positive correlation (r= 0.63) in males [43]. A reduction in caudate volume after three years of the illness onset was also observed in a sample of 109 early onset schizophrenia patients (mean age= 28.4 years) treated with antipsychotics, both typical and atypical, comparing with 76 healthy volunteers [44]. 

Lateral ventricle (LV) changes were investigated in the lengthiest study included in the review. It covered a period of ten-year follow-up in chronic patients with schizophrenia (mean age= 37.5 years) taking haloperidol. Authors concluded that changes in LV were not associated with daily haloperidol exposure [45]. Puri and colleagues obtained similar results in a sample of FEP patients (mean age=28.5 years) treated with different types of FGAs and SGAs. Changes in LV size were not associated with total duration of treatment or with total cumulative dose over 30 weeks [46].

### Effect of Different Antipsychotic Treatments

The differential effects of FGAs and SGAs, which have different profiles of affinity for dopamine D_2_ receptors on brain structure have attracted much interest since medications are taking chronically. 

In 1999, Corson *et al*. published a two-year longitudinal MRI study with 23 male patients (mean age 25.57 years) with diagnoses in the schizophrenia spectrum. During the two-year period, “mean basal ganglia volume of patients receiving predominantly FGAs increased, while the opposite was observed for patients receiving mostly SGAs” [47]. Basal ganglia volumes have been also examined in a longitudinal, open-label study conducted on “drug-naïve first-episode schizophrenia before and after short-term treatment with either” [48] a FGA and SGA drug. Nineteen FEP patients (mean age 26 years) and 19 matched healthy controls were included. From the patient group, 16 were antipsychotic drug-naïve, and three minimally medicated first-episode schizophrenia patients. Overall, typical (zuclopenthixol) and atypical (risperidone) “medication groups did not differ significantly with respect to volume changes after three months of low-dose treatment” [48] in basal ganglia volumes. “Nevertheless, when medication groups were examined separately, a significant volume increase in the putamen was detected in the risperidone group. Furthermore, a time by gender effect was found in the accumbens nucleus, which was explained by an increase in accumbens volume in the male patients and a decrease in female patients. There were no significant main or interaction effects in the FGA group” [48] treated with zuclopenthixol.

Garver and colleagues (2005) examined cerebral GM in 19 patients (mean age 33 years) “with schizophrenia before and after 28 days of treatment with FGAs (haloperidol; N=6) or SGAs (risperidone and ziprasidone; N=13)” [49] medication. During treatment with the atypical medication, “cerebral cortical GM of 13 patients with schizophrenia expanded” [49]. However, the group of six patients that received “haloperidol, as well as the seven control subjects, showed no change in cortical GM volumes [49]” at the time of reassessment. 

In a “longitudinal, randomized, controlled, multisite, double-blind study” [50] , Liberman and colleagues (2005) studied the antipsychotic drug effects on brain morphology in FEP. “Patients were randomized to double-blind treatment with olanzapine, 5 to 20 mg/day, or haloperidol, 2 to 20 mg/day for up 104 weeks. MRI assessments were performed at weeks 0 (baseline), 12, 24, 52, and 104” [50]. One hundred sixty-one patients (mean age 23.85 years) received a baseline and at least one follow-up MRI measure. Authors found that haloperidol was associated with significant reductions in GM volume, whereas olanzapine failed to show this association. “A matched sample of healthy volunteers (N=58) examined contemporaneously showed no change in GM volume” [50]. Authors then hypothesized that these “differential treatment effects on brain morphology could be due to haloperidol-associated toxicity or greater therapeutic effects of olanzapine” [50].

McCormick *et al*., (2005) investigated whether SGAs differed from FGAs “in their effect on anterior cingulate volume change over time” [51]. They studied 31 antipsychotic-naïve subjects (mean age 24.8 years) diagnosed with schizophrenia. The average follow-up period was 3 years (range 2 to 5 years). They found that increased FGA “exposure over time was correlated to increased anterior cingulate volume over time” [51], whereas increased SGA exposure was correlated to the opposite effect and the cingulated volume decreased [51]. 

In 2008, Crespo-Facorro and colleagues studied the “effects of risperidone (N=16), olanzapine (N=18) and low doses of haloperidol (N=18) in brain volume changes during one-year follow-up period in a large and heterogeneous sample of first episode schizophrenia spectrum patients” [52] (mean age 29.76 years). They found “a significant increase in lateral ventricles in patients treated with risperidone. Patients exposed to atypical drugs (olanzapine and risperidone) exhibited a decrease in caudate nucleus volume“ [52]. 

In 2011, Ho and colleagues explored whether FGAs, non-clozapine SGAs, and clozapine might “have differential effects on brain volumes in schizophrenia” [26]. They studied 211 patients with schizophrenia (mean age 26.3 years) “who underwent repeated neuroimaging beginning soon after illness onset” [26]. The sample included 674 MRI scans “covering a mean follow-up period of 7.2 years (range, 1.9–14.0 years), and inter-scan intervals were approximately three years” [26]. Higher FGA doses “were associated with smaller total cerebral GM and frontal GM volumes. Higher doses of non-clozapine SGAs were associated with lower frontal and parietal GM volumes, and higher clozapine doses were associated with smaller total cerebral and lobar GM volumes. For WM volumes, higher non-clozapine SGA doses were significantly associated with larger parietal WM volumes. Higher clozapine doses were associated with larger sulcal cerebrospinal fluid (CSF) volumes and smaller subcortical (caudate, putamen, and thalamic) volumes” [26].

Variations in cortical thickness in relation with different drugs have been also investigated. In 2012, Roiz-Santiañez and colleagues, with the same sample used by Crespo-Facorro *et al*., (2008), examined cortical thickness changes but they did not find significant effects of time, treatment group or group-by-time interaction for any of the cortical thickness variables evaluated [53]. Recently, van Haren *et al*. (2011) examined cortical thickness and change in cortical thickness on a vertex-by-vertex basis across the cortical mantle. They performed a five-year follow-up MRI study that included 96 schizophrenia patients (mean age: 32.22 years) and 113 healthy controls (mean age: 35.28 years). “Significant correlations between medication variables and cortical thickness change” [33] showed that, for FGAs, correlations were negative (i.e. higher intake was associated with more pronounced decreases in cortical thickness), whereas correlations with SGAs were positive (i.e., higher intake was related to less decreases in cortical thickness) [33].

### Effect of Antipsychotic Medication Switch

Two studies have examined changes in brain structure after a change in medication. A first study performed by Scheepers *et al*., in 2001 examined “the effect of clozapine on caudate nucleus volume in schizophrenia patients previously treated with FGAs” [54]. Twenty-six patients (mean age: 35.23 years) participated in this open design study. Treatment with clozapine resulted in a decrease in caudate volume. However, no differences in caudate volume changes were found between responders and non-responders to clozapine [54]. In other study, Lang and colleagues (2004) examined 37 patients with schizophrenia. From them, those who were receiving FGAs (N=10) or those taking risperidone with “limited response (N=13), were switched to treatment with olanzapine (N=23). Patients receiving risperidone and exhibiting a good response (N=14) continued treatment with the same treatment” [55]. Authors observed that “olanzapine reversed putamen and globus pallidus enlargement induced by FGAs but did not alter volumes in patients previously treated with risperidone” [55].

## DISCUSSION 

The present review article investigated the effect of antipsychotic medication on brain structural changes found in patients with schizophrenia. Forty-one longitudinal MRI studies with antipsychotic medication administration were included with a final database of 1391 schizophrenia patients and 1135 controls. The influence of antipsychotic medication in relation to longitudinal brain changes remains inconclusive. There was a large inconsistency among the results from the different studies and also a great heterogeneity with regard to sample size, follow-up duration, antipsychotic medication type and dose, MRI processing analyses or illness duration. This makes not possible to achieve clear conclusions regarding the effects of antipsychotic medication on brain structure.

The analyses of the relationship between the degree of exposure to antipsychotic medication and brain morphometry change have also led to mixed results. Overall, most of the included studies did not find a linear relationship between the degree of exposure and progressive brain changes. Although some studies found different associations, these were inconsistent. Whereas some studies suggested associations between antipsychotic medication exposure and a decrease in global grey matter [21], others found regional volume loss to be dose-dependent. Massana and colleagues (2005) reported that treatment with risperidone was associated, in contrast to the findings for other SGAs [39, 56], with increases in the caudate volume. Keshaban and colleagues (1994) showed a caudate increment across time but using FGAs [37]. Ebdrup and colleagues (2011) directly correlated quetiapine doses with hippocampal volume loss but they found an inverse correlation between quetiapine doses and striatal volume loss [41]. 

The different effects of FGAs and SGAs on brain morphometry gave also contradictory results. It is been suggested that SGAs could delay, prevent or even reverse cortical loss [57, 58]. Interestingly, Lieberman and colleagues (2005) observed that the differential effects on brain structure of FGAs and SGAs lost significance across time [50]. The effects of FGAs and SGAs in subcortical structures remain also ambiguous. Corson *et al*. (1999) reported an increment in basal ganglia volume in patients receiving predominantly FGAs, “while the opposite was observed for patients receiving mostly SGAs” [47]. However, Glenthoj *et al*., (2007) reported no differences with respect to basal ganglia volume changes after three months of low-dose treatment with FGAs and SGAs [48]. A recent literature review has conclude that the assumed “increase of basal ganglia volumes in patients with schizophrenia appears to be related to the D2 blockage due to antipsychotic administration, which is particularly relevant for the FGAs and chronic administration” [59]. Some authors have also suggested “that gross striatal morphological changes may represent candidate biomarker for the risk and outcome of the illness” [60].

A volume reduction has been associated with a medication switch in both studies focused in medication switch to SGAs in patients who had been previously treated with FGAs [54, 55]. It is been suggested that this reduction might represent normalization rather than atrophy. 

The mechanisms of antipsychotics action on brain structure can be better understood from animal and *in vitro* studies. Some authors described similar effects for typical and atypical agents. Thus, in a recent *in vivo *study, Vernon and colleagues (2014) “developed a rodent model that uses clinically relevant doses and serial magnetic resonance imaging (MRI), followed by postmortem histopathological analysis to study the effects of antipsychotics on brain structures” [61]. This longitudinal study found that chronic (8 weeks) antipsychotic treatment results in altered brain morphology in rodents. “The exposure to both haloperidol and olanzapine resulted in significant decreases in WBV (6% to 8%)” [61] and in the cerebral cortex volume (8% to 12%) compared with the control group. “Hippocampal, corpus striatum, lateral ventricles, and corpus callosum volumes were not significantly different from control subjects, suggesting a differential effect of antipsychotics on the cortex” [61]. Similarly, Dorph-Petersen *et al*., in 2005, found “that macaque monkeys chronically treated (2.5 years) with haloperidol or olanzapine showed an approximate 10% reduction in brain weight relative to monkeys receiving placebo” [9]. However, Andersson *et al*., (2002) reported “different effects of FGAs and SGAs in brain morphometry. In this case, haloperidol exposure was associated with a significant increase in caudate–putamen volume and olanzapine exposure with a reduction” [62] in these structures. Evidence from animal and cell biology studies have suggested that dopamine “receptor blockade might influence cell proliferation by modulating brain growth factors” [15] and cellular morphology [14]. Nevertheless, some “caution needs to be exerted when extrapolating results from animals studies to humans” [61].

Previous studies have suggested that “dynamic alterations in gray matter structure can occur very rapidly within a time range of a single week” [63]. A recent “ *in vivo* multimodal pharmaconeuroimaging study in seven neuroleptic-naive healthy male volunteers undergoing a haloperidol challenge (5 mg per 70 kg of body weight, intravenous application) found reversible striatal volume changes and structural-functional decoupling in motor circuits within hours” [64]. Therefore, changes in brain volumes could occur at the very beginning of antipsychotic treatment and seem to be nonlinear [65]. Hence, the follow-up periods of the articles included in this review (range from 4 to 500 weeks) could be considered too long to detect potential effects of antipsychotic medication on brain structure. 

There is not, to the best of our knowledge, any longitudinal MRI study conducted on untreated schizophrenia patients. All available studies were conducted on chronic medicated patients, or FEP drug-naive patients that were treated since the illness onset, and thereby they were taking medication during the follow-up. Therefore, it remains unclear whether potential structural changes are due to the ongoing illness process or they may be mediated by antipsychotic medication and other confounder factors, such as illness duration, changes in clinical severity, cognitive deficits or substance abuse. In this context, larger ventricles and decreased prefrontal volumes have been correlated with worse outcome [66]. Therefore, clinical heterogeneity, small sample sizes, and the effect of other confounding factors such as poor nutrition [67], diminished social and environmental stimuli [68], alcohol, tobacco or cannabis consumption [69-71], and lifestyle, like physical exercise [72, 73] may account for discrepancies between studies. 

“Differences in the methodology may also account for inconsistencies between investigations. Reliability in MRI-derived automated morphometric measures can be influenced by several sources of variance” [74]. Therefore, reliability can be affected by subject-related factors, such as hydration status [75], instrument-related factors, such as field strength, scanner manufacturer, imaging magnetic gradients and pulse sequence [76], and data processing-related factors, including the software package and version and the parameters used in the analysis [77-79].

Although we choose to group the studies in three categories: effect of different antipsychotic treatment, effect of switch, association with antipsychotic medication exposure, we acknowledge that other choices could have been made (comparing studies with FGAs vs SGAs, or according to the stage of illness (first episode vs chronic patients) or according to brain areas or tissue).

In conclusion, current longitudinal MRI studies do not provide a consistent description of the effects of antipsychotic treatment on the pattern of brain changes over time in schizophrenia patients. Further short- and long-term studies longitudinal studies are warranted to verify whether antipsychotics may produce brain structural changes or whether FGAs and SGAs may differentially affect brain morphometry.

## Figures and Tables

**Table 1:** Overview of studies included in the review.

		Healthy Controls		Patients							
Type	Study	N	Age Years, mean (SD)	N	Age Years, mean (SD)	Status	Medication	CPZ E mean (SD)	MRI Software and Analysis Type	Outcome Variables	Follow-up Duration (weeks) mean (SD)
Association	Arango C *et al*. 2012						QUE, OLA, RIS, ZIP, ARI, others	Cumulative: 165658 (90011) mg	In house software Automated ROI	WBV, GM, WM, CSF	104
Association	Boonstra G *et al*. 2011	20	28.0 (5.6)	16	28.8 (6.9)	FEP	OLA, RIS, QUE	Cumulative: 42827.5 (43192.5) mg	Automated and manually traced ROI	ACCU, PUT	52
Association	Cahn W *et al*. 2002	36	24.5 (5.8)	34	26.2 (5.31)	FEP	FGA, SGA	Cumulative: 103675 (48135) mg	In house software Semi-automated ROI	BV, GM, LV	50.8 (4.4)
Association	Chakos MH *et al*. 1994	10	30.5 (4.9)	29	25.2 (6.3)	FEP	FGA, SGA	n.s	Manually traced ROI	CAU, LV	72
Association	Cobia DJ *et al*. 2012	20	30.4 (12.8)	20	31.9 (11.1)	Chronic	FGA, SGA	FGA: 0.9 (2.9) dose-year SGA: 3.5 (3.4) dose-year	FreeSurfer Automated ROI	FRO, TEM CT	104
Different treatments	Corson PW *et al*. 1999	---	---	23	22.6 (6.2)	Chronic	FGA	FGA: 5.6 (6.%) dose year SGA: 5.3 (7.8%) dose year	BRAINS Manually traced ROI	CAU, PUT, ACU	112.8 (21.8)
Different treatments	Crespo-Facorro B *et al*. 2008	38	Matched	18 16 18	29.8 (7.9) 28.0 (5.1) 25.0 (6.0)	FEP	HAL RIS OLA	244.1 mg/d 183.6 mg/d 289.0 mg/d	BRAINS2 Semiautomated ROI	WBV, GM, WM, LV, CAU	54.6 (4.6)
Different treatments	DeLisi LE *et al*. 2004	20	25.5	26	26.8 (7.1)	FEP	FGA, SGA	n.s	ANALYZE Semi-automated	LV	520
Association	Edbrup BH *et al*. 2011	28	28.4 (6.0)	13: low dose	26.2 (5.7)	FEP	QUE	< 430.4 mg/d	SPM5 ROI mask	ACCU, CAU, PUT, HP	36
				9: high dose	27.8 (5.1)			≥ 430.4 mg/d			
Different treatments	Glenthoj A *et al*. 2007	19	27.5 (5.3)	16	25.9 (5.1)	FEP	ZUCLO, RIS	Cumulative: ZUCLO: 168 mg RIS: 100-240 mg	Manually traced ROI	CAU, PUT, ACU	12
Association	Frazier JA *et al*. 1996	8	15.1 (2.3)	8	15.4 (3.1)	Chronic	CLOZ	400 (128.9) mg/d	Manually traced ROI	ACCU, CAU, PUT, HP	104
Different treatments	Garver DL *et al*. 2005	7	29.0 (9.0)	19	33.0 (12.0)	Mixed	RIS, ZIP, HAL	RIS: 400 mg/d ZIP: 300 mg/d HAL: 350 mg/d	In house software Automated ROI	GM	4
Association	Goghari VM *et al*. 2013	26	20.9 (2.1)	19	18.9 (3.6)	FEP	RIS (16), QUE (3)	144 mg/d	FreeSurfer Automated ROI	PreFRO CT	8
Association	Gur RE *et al*.1998	17	31.9 (8.9)	20 drug-naive	27.8 (8.2)	Mixed	RIS, CLOZ, CPZ, HAL, FLUP, LOX, MESO, MOLI, THIO, TRIFLU	259.9 (165.6) mg/kg	Manually traced ROI	CSF, BV, FRO and TEM lobes	122.5 (51.7)
				20 medicated	30.6 (7.7)						
								513.3 (224.0) mg/kg			
Association	Heitmiller DR *et al*. 2004	14	26.7 (11.3)	14	26.3 (6.8)	FEP	SGA	7.4 (5.5) dose-year	BRAINS2 Semiautomated ROI	CAU	Patients: 120.8 (53.2) Controls: 129.6 (48.4)
Association	Ho BC *et al*. 2003	23	26.9 (5.3)	72	24.5 (4.67)	FEP	FGA, CLOZ, SGA	n.s	BRAINS2 Semiautomated ROI	BV, LV, CSF, cerebellum, FRO, TEM, PAR	Patients: 171.1(77) Controls: 176.3 (83.2)
Association; different treatments	Ho BC *et al*. 2011	---	---	70 high dose 70 intermediate dose 71 low dose	26.3 (7.6)	FEP	FGA, CLOZ, SGA	924.4 mg/d 391.7 mg/d 111.5 mg/d	BRAINS2 Semi-automated ROI	WBV, GM, WM, TEM, FRO, PAR , LV, CSF, cerebellum, PUT, CAU, THA	374.4 (202.8)
Association	Keshavan MS *et al*.1994	---	---	11	n.s	FEP	HAL, FLUP, PERH	112 (60) mg/d	NIH Image Manually Traced ROI	CAU, pre FRO CT, GM, WM, WBV.	43.6 (31.1)
Switch treatments	Lang DJ *et al*. 2004	23	23.2 (7.4)	10	35.3 (8.8)	Chronic	FGA, RIS	170 (64) mg/d	NIH Image Manually Traced ROI	CAU, PUT, PALL	56 (17.1)
Different treatments	Lieberman JA *et al*. 2005	62	25.5 (4.1)	79	24.1 (4.6)	FEP	HAL, OLA	HAL: 135-500 mg/d OLA: 100-1000 mg/d	Automated and manually traced ROI	WBV, GM, LV	52
Association	Massana G *et al*. 2005	---	---	11 drug-naive	23.0 (4.0)	FEP	RIS	605 mg/d	Automated ROI	PUT, CAU, PALL, AMIG	12
Association	McClure RK *et al*. 2008	---	---	10 medicated	36.7 (7.7)	Chronic	SGA	400 mg/d	ITK-SNAP Semi-automated ROI	CAU, FRO and TEM GM, WM, CSF, LV	12
Different treatments	McCormick L *et al*. 2005	18	30.5 (6.9)	31	24.8 (5.9)	FEP	SGA, FGA, both	n.s	BRAINS2 Manually traced ROI	Cingulate volume	104-156
Different treatments; association	Molina V *et al*. 2005	11	28.4 (6.2)	17 drug naive	25.6 (4.0)	FEP	RIS	500 (200) mg/d	In house software Automated ROI	GM, WM	104
				12 medicated	31.0 (5.9)	Chronic resistant	CLOZ	410 (339) mg/d			
Association	Nakamura M *et al*. 2007	26	22.9 (3.8)	17	24.3 (5.8)	FEP	SGA*	266.3 mg/d	In house software Manually traced ROI	NCGM, CSF, GM, WM, LV	78
Association	Okugawa G *et al*. 2007	10	31.9 (5,1)	10	31.6 (6.3)	Chronic	OLA	357.5 mg/d	BRAINS2 Semi-automated ROI	CAU	26.6 (8.6)
Association	Puri BK *et al*. 2001	12	27.9 (6.1)	24	28.5 (8.4)	FEP	3 drug-naive, 4 RIS, 5 FGA	Cumulative: 68365.9 (53879.5) mg	semi-automated computerised technique	LV	30.9 (6.2)
Association	Reig S *et al*. 2009	34	15.2 (1.4)	21	15.7 (1.7)	FEP	QUE, OLA, RIS, CLOZ, others	n.s	In house software Automated ROI	GM, WBM, CSF	104
Different treatments	Roiz-Santiañez R *et al*. 2012	52	28.8 (7.4) 27.3 (5.9) 29.6 (6.1)	45	Matched	FEP	HAL (18) OLA (18) RIS (16)	244.1 mg/d 282.0 mg/d 183.6 mg/d	BRAINS2 Semi-automated ROI	CT	52
Association	Roiz-Santiañez R *et al*. 2012	70	Matched	93	28.3 (7.8)	FEP	HAL (11), OLA (19), RIS (22), QUE (16), ZIP (12), ARI (11), no antipsychotic (2)	224.9 mg/d	BRAINS2 Semi-automated ROI	SG	54.9 (5.3)
Association	Roiz-Santiañez R *et al*. 2014	76	Matched	109	28.4 (7.8)	FEP	HAL(19), OLA (19), RIS (20), QUE (17), ZIP (18), ARI (16)	Cumulative: 99149 (86605) mg	BRAINS2 Semi-automated ROI	WBV, GM, WM, CAU, CSF, LV, THA	156
Association	Saijo T *et al*. 2001	12	37.1 (4.2)	18	37.5 (8.9)	Chronic	HAL	2,075 (1080) mg/kg/d	NIH Image Manually traced ROI	LV	520
Switch treatments	Scheepers FE *et al*. 2001	---	---	29	35.2 (10.3)	FEP	Swiching from FGA to CLOZ	345.6 (63.4)	ANALYZE Manually traced ROI	WBV, CAU	24
Association, different treatments	Sporn AR *et al*. 2003	43	14.8 (2.2)	39	15.0 (2.3)	FEP	CLOZ, SGA, CLOZ + FGA, CLOZ +SGA	n.s	Fully automated technique	WM, WBV, LV	176.8 (72.8)
Association	Takahashi T *et al*. 2009	26	25.6 (9.1)	23	21.6 (3.5)	FEP	SGA, FGA	n.s	Dr View Manually traced ROI	WBV	105 (39.2)
Association	Tauscher-Wisniewski S *et al*. 2002	10	29.4 (8.6)	15	23.0 (6.2)	FEP	FGA, SGA	Cumulative: 15093 (387) mg	BrainImage Manually traced ROI	CAU	260
Association	Tauscher-Wisniewski S *et al*. 2005	37	25.8 (6.2)	14	33.6 (3.7)	FEP	QUE	395.2 (103.2) mg/d	BrainImage Manually traced ROI	CAU	12
Association	Taylor S *et al*. 2005	11	26.8 (6.6)	11	34.7 (12.4)	FEP	FGA, SGA	n.s	Amira FreeSurfer Automated ROI	CAU, PUT	4
Association	Van Haren N *et al*. 2008	113	35.3 (12.3)	96	32.2 (11.1)	Chronic	FGA, CLOZ, SGA	Cumulative: FGA: 1828 (1238) mg SGA: 1719 (1949) mg CLOZ: 126615 (42247) mg Swiched and used: typical:366 (451) mg Atypical: 819 (726) mg CLOZ: 80932 (48811) mg	In house software Automated ROI	WBV, GM, WM, LV	260
Association, different treatments	Van Haren N *et al*. 2011	113	35.3 (12.3)	96	32.2 (11.1)	Chronic	FGA, CLOZ, SGA	Cumulative: FGA: 1828 (1238) mg SGA: 1719 (1949) mg CLOZ: 126615 (42247) mg Swiched and used: FGA:366 (451) mg SGA: 819 (726) mg CLOZ: 80932 (48811) mg	FreeSurfer Automated ROI	CT	260
Association	Whitworth AB *et al*. 2005	20	31.5 (4.9)	21	25.0 (4.7)	FEP	n.s.	n.s.	Manually traced ROI	WBV	104-208
				17	28.4 (4.0)	Chronic					
